# Analysis of suicide statistics and trends between 2011 and 2021 among Korean women

**DOI:** 10.4069/kjwhn.2023.12.14.1

**Published:** 2023-12-28

**Authors:** Hyunjung Jang, Seokmin Lee, Sanghee Park, Bobae Kang, Hyunkyung Choi

**Affiliations:** 1 Department of Nursing, Catholic Kkottongnae University, Cheongju, Korea; 2 Statistical Research Institute, Statistics Korea, Daejeon, Korea; 3 Vital Statistics Division, Statistics Korea, Daejeon, Korea; 4 College of Nursing & Research Institute of Nursing Innovation, Kyungpook National University, Daegu, Korea

**Keywords:** Mortality, Suicide, Women

## Abstract

**Purpose:**

This study aims to analyze the number of suicide deaths in women, trends in suicide mortality, characteristics of suicide by age, and outcomes of suicide means over the past decade (2011–2021) in South Korea.

**Methods:**

Using cause of death data from Statistics Korea, an in-depth analysis of Korean women’s suicide trends was conducted for the period of 2011–2021.

**Results:**

In 2021, women’s suicide death in Korea was 4,159, a rate of 16.2 per 100,000 population. The rate increased by 1.4% from the previous year. Since 2011, women’s suicide rate has been on a steady downward trend, but since 2018, it has been on the rise again. Suicide rates among women in their 20s and 30s have increased, especially since the coronavirus disease 2019 pandemic, and suicide rates among women over 70 years remain high. As compared to 2011, pesticide poisoning and hanging among the means of suicide have decreased significantly, while drug and carbon monoxide continue to increase.

**Conclusion:**

Suicide rates for Korean women in their 20s and 30s have increased significantly in recent years, and those for women over 70 years remain high. Therefore, it is necessary to investigate the causes and establish national policies for targeted management of these age groups, which contributes significantly to the rising suicide rate among Korean women.

## Introduction

In 2021, the number of deaths in South Korea (hereafter, Korea) reached 317,680, a 4.2% increase from the previous year, reaching its highest level in the past decade [1]. The suicide rate was 26.0 (the number of deaths per 100,000 population), up 1.2% from the previous year [1]. Moreover, Korea recorded the highest level, 2.1 times higher than the Organization for Economic Co-operation and Development (OECD) average of 11.1 (the number of deaths per 100,000 standard population) [1]. In particular, it was reported that the suicide rate of women has been continuously increasing since 2017 [2]. To reduce suicide, Korea has been establishing the National Suicide Prevention Master Plan every 5 years since 2004 as a national suicide prevention strategy [3], but it has been ranked first since 2018 among OECD countries [4].

Statistics Korea reports the results of cause of death statistics every year, but it analyzes and describes suicide as one of several causes of death. According to the 2023 Suicide Prevention White Paper published annually by the Ministry of Health and Welfare and the Korea Foundation for Suicide Prevention, the number of suicide attempts by women is 1.4 times higher than that by men [5]. Women are reported to be more vulnerable to suicide because they have lower socioeconomic levels than men [6], such as income, education, and employment, and a higher prevalence of mood and anxiety disorders, including depression [7]. Therefore, a detailed approach that considers the changing trends in suicide rates and differences in suicide rates by gender and age is needed to develop policies to effectively reduce suicide rates.

Thus, this study aims to update Korean women’s suicide death statistics, suicide rate trends, gender- and age-specific suicide characteristics, and suicide means over the past 10 years from 2011 to 2021. The trends identified can provide basic data for policy establishment for suicide prevention at the present time when women face increasing vulnerable factors.

## Methods

**Ethics statement:** This study was a secondary analysis of existing mortality data and did not require Institutional Review Board approval or informed consent.

### Data sources

This study used cause-of-death statistics from Statistics Korea to analyze trends in suicide deaths among Korean women. The cause-of-death statistics were compiled based on death certificates collected by Statistics Korea. In this study, microdata on cause of death statistics from 2011 to 2021 were analyzed through the MicroData Integrated Service of Statistics Korea [8]. This data includes demographic characteristics of the deceased and detailed causes of death, including external factors (accidents, etc.). To increase the accuracy of causes of death, Statistics Korea reflects administrative records from institutions such as the National Cancer Center, Health Insurance Review and Assessment Service, and the National Institute of Scientific Investigation.

### Study variables

The variables used in this study were total deaths, suicide deaths, suicide death rates, female mortality rates, and means of suicide.

### Definition of terms

#### Suicide deaths

This refers to the act in which an individual, with the intention of causing their own death, deliberately ends their own life using any means or method (in cases where parents commit suicide with young children, the death of young children is classified as homicide rather than suicide) [1].

#### Age-standardized suicide death rate

The death rate was adjusted to account for the impact of age structure on mortality levels, allowing for mortality comparisons between populations with different age distributions [1].

#### Suicide means

Among the classification codes according to the Korean Standard Classification of Diseases, the codes of suicide means are further subdivided (X60–X84). They are as follows: hanging (X70); fall (X80); carbon monoxide (X67.0–X67.4); pesticides (X68); drowning (X71); other and unspecified gases (X67.8–X67.9); drugs (X60–X64); and others (X65, X66, X69, X72–X79, X81–X84) [1].

### Statistical methods

Data on all deaths were used as the statistical data and analyzed using descriptive statistics and calculations according to the formula based on the definition of terms.

## Results

In 2021, the number of suicide deaths in Korean men and women reached 13,352, an increase of 157 (1.2%) compared with the previous year, with a suicide rate of 26.0 per 100,000 population (Table 1). The suicide rate peaked at 31.7 per 100,000 population in 2011 and then showed a declining trend for 6 years. However, in 2018, the suicide rate increased again to 26.6 per 100,000 population, temporarily dropping to 25.7 in 2020, but rebounding to 26.0 in 2021.

### Trends in suicide rates by gender

Examining the trend in suicide rates by gender, in 2021, the number of suicide deaths among women reached 4,159, with a suicide rate of 16.2 per 100,000 population, a 1.4% increase from the previous year (Table 1). In the “age-standardized suicide death rate,” which eliminates differences in age structures among nations, women’s suicide rate increased by 2.1% compared with the previous year, indicating a larger increase in suicides in women. The gender-specific suicide rate difference, which was 23.2 per 100,000 population in 2011, decreased to 19.7 in 2021, signifying a gradual increase of suicide among women and a narrowing gender gap in suicide rates.

### Trends in suicide rates by age

**Figure 1.**
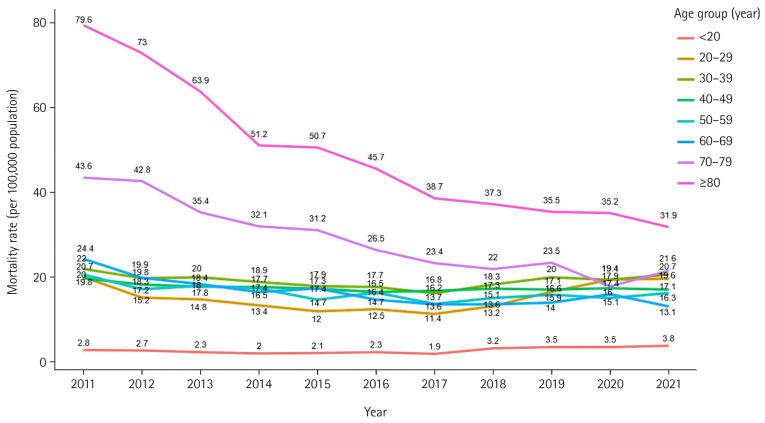
Women’s suicide rate by age group (South Korea, 2011–2021).

Over the past 10 years (2011–2021), the suicide rate for all women has shown an overall downward trend (Figure 1). Compared with 2011, the age-specific suicide rate in 2021 decreased in all age groups over 20 years, with the largest decreases in the 70–79 and over 80 years age groups at 50.5% and 60.0%, respectively. However, compared with 2020, the age-specific suicide rates in 2021 increased to 8.4%, 1.2%, and 7.0% for the under 20, 20–29 years, and 30–39 years age groups, respectively. Since 2018, suicide rates have increased significantly in the 20–29 and 30–39 years age groups. Suicide rates among young women in their 20s and 30s increased markedly in 2020 and 2021, during the coronavirus disease 2019 (COVID-19) pandemic (Table 2).

### Trends in suicide rates by means

**Figure 2.**
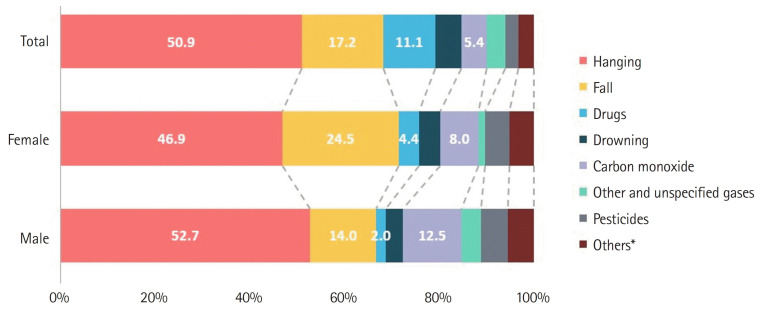
Composition of means of suicide (South Korea, 2019–2021). ^*^Others: X65 (alcohol, organic), X66 (solvents, halogenated hydrocarbons, their vapors), X69 (other and unspecified chemicals and noxious), X72–X79 (handgun; rifle, shotgun, larger firearm; other and unspecified firearm; explosive material; smoke, fire, flames; steam, hot vapors, hot objects; sharp object; blunt object), X81–X84 (jumping or lying before moving object; crashing of motor vehicle; other specified means; unspecified means).

In the recent 3-year period (2019–2021), the most common means of suicide for women were hanging (46.9%), falling (24.5%), and carbon monoxide (8.0%) (Figure 2). Drugs (sedative-hypnotic drugs, psychotropic drugs, etc.) and carbon monoxide poisoning are two of the means of women’s suicide that have steadily increased from 2011 to 2021. Specifically, drug use increased 220.5% and carbon monoxide increased 134.3%, whereas pesticide poisoning and hanging decreased significantly to 75.0% and 28.8%, respectively (Table 3).

## Discussion

The gender ratio of suicide rates has decreased, indicating a relatively higher increase of suicide deaths in Korean women compared with men [1]. Furthermore, in 2021, the women’s suicide death rate reached 16.2 per 100,000 population, a significant increase of 1.4% from the previous year [1].

The foreign press concluded that the high suicide rate of young Korean women influenced this trend, noting that Korea’s suicide rate, which had been on the decline over the past decade, began to increase again in 2018, and was the highest among OECD member countries [9]. In Korean society, women are expected to take on the dual roles of household chores and childcare, while also participating in the workforce. This contradictory situation, coupled with discrimination in the workplace, contributes to an increase in suicide among Korean women [9]. Analyzing the proportion of non-regular workers in 2022, women accounted for 55.2%, which is 10.4% higher than men, and the proportion of non-regular men and women workers by age group was highest in those 60 years or older (31.3%), followed by 50–59 years (21.1%) and 20–29 years (17.3%) [10]. During the COVID-19 pandemic, the service industry was particularly devastated, and naturally, young women were inevitably affected [10]. Even before the pandemic, however, Korean women have been a vulnerable group regarding employment and self-reliance, compared to their male counterparts. During the 2008 financial crisis in Korea, non-regular workers were converted to part-time workers, and women occupied more of the lower-income portion of the workforce; women became assistants and worked mainly in the service sector [11]. Women are significantly impacted when socioeconomic crises worsen. Pervasive gender discrimination, the instability of female employment, violence against women, the burden of balancing work and family life, and the feminization of poverty have been pointed out as preexisting factors that deteriorate the quality of life of women in Korea [12]. Specifically, the degradation of the quality of life among young Korean women is attributed to factors such as a lack of career advancement opportunities, gender discrimination, biases, relative deprivation compared to men, economic weakening owing to job disparities or lack of promotions, and domestic violence [10]. As a countermeasure, suicide prevention policy should focus on changing the overall social atmosphere, gender-discriminatory appearance standards, and the culture that tolerates sexual abuse using hidden cameras. Violence against women in the labor market, such as sexual violence, dating violence, sexual contempt, and bullying should not be condoned [13]. Enforcing workplace policies that eliminate discrimination can foster women’s independence and hope. Additionally, policies that allow for career breaks during marriage or childcare will enable women to be equal members of society and participants in the labor market, as individuals who work together for social development.

Since 2011, the suicide rate for Korean women aged 70 years and older has continued to decline, and the decline is significant. While this is a positive phenomenon, there is still a high rate of suicide among this age group compared with women in other age groups. In this regard, greater attention should be given to older women living alone, especially as Korea has the largest number of poor seniors among OECD countries [14], and economic differences owing to insufficient income and gender wage gaps have emerged as social problems [14]. The suicide rate owing to physical disease problems is higher than that of other age groups [6] and the subsequent economic burden is also a major cause of the high suicide rate [6]. Despair, loneliness, and the death of a meaningful person or spouse can increase suicide rates among older adults [5,15]. This means that as women’s lifespan increases [16], the number of older people living alone increases, and the proportion of older women at risk of suicide also increases.

In 2020, 35.1% of households with a head of household age 65 years or older were single-elderly households. Of these, 44.1% were in their 70s, and 71.9% were women [17]. These older adults living alone rated their subjective health negatively, had poorer overall healthcare practices than all older adults, and 44.6% were self-supporting [17], indicating both economic poverty and healthcare vulnerability. Korean women experience a 1.5 to 2 times higher prevalence of depression than men [7], a systematic review of suicide among older Koreans living alone reported a high proportion of women aged 70 years and older living alone and found that higher levels of depression and lower levels of social support were associated with a higher risk of suicide [18].

According to a previous study [18], depression had the strongest effect on suicidal ideation in older adults, and higher levels of depression were associated with higher levels of suicidal ideation. However, social support from the family had a moderating effect on the relationship between depression and suicidal ideation. This indicates that older people living alone and with limited family support are more likely to be depressed and suicidal. Therefore, there is a need to screen older women for depression and provide them with community-based social support. Currently, Korea is conducting various types of projects for vulnerable populations through integrated health promotion programs. Nurses can promote mental health and counteract the vulnerability among older women living alone, especially through the effective implementation of home-visiting health care programs [19].

There are multiple factors to consider in the recent increase in suicide rates found among young women in their 20s and 30s, e.g., a steep increase starting in 2018 and a 7.0% increase from 2020 to 2021 in women in their 30s. The relationship between individual unhealthy behaviors, such as smoking and drinking, and suicide risk; and with environmental vulnerability during the COVID-19 pandemic are worth examining. Current smoking prevalence is the percentage of people who smoked five or more packs of cigarettes in their lifetime and are currently smoking. Among Korean men, the current smoking prevalence has continued to decline from 47.3% in 2011 to 31.3% in 2021, whereas it remained similar among women, from 6.8% in 2011 to 6.9% in 2021 [20]. In particular, Korean women in the 20–40 years age group showed an increasing trend in smoking rates compared with other age groups [21].

Meanwhile, monthly drinking rates (drinking at least once a month in the past year) for Korean women in 2021 were 60.6% for 19 to 29-year-olds and 56.9% for 30 to 39-year-olds, with more than half of the women reporting daily drinking [22]. In the 2021 data, women aged 30–39 years reported the highest rate of high-risk drinking, at 13.2%, followed by those aged 19–29 and 40–49 years at 10.7% [23]. A previous study [24] that analyzed data from the Korean National Health and Nutrition Examination Survey found that suicidal ideation was 1.56 times higher among alcohol abusers than among moderate drinkers, and 1.34 times higher among current smokers than among nonsmokers. Furthermore, compared with nonsmokers and moderate drinkers, current smokers and alcohol abusers had a 2.13 times higher risk of suicidal ideation and a 3.81 times higher risk of suicide attempts [23]. Given that smoking and drinking in women have been reported to be significantly associated with the risk of suicide death [25], the increasing prevalence of smoking and risky drinking in young women may be related to the increasing suicide rate. Therefore, efforts should be made to increase health-promoting behaviors in women.

After the COVID-19 pandemic, the issues have been managed through “COVID-19 Women’s Employment Crisis Recovery Measures” [26], “Elderly Welfare Policy” [27], but support has been concentrated on women in their 30s and 50s, women who are responsible for caring, and older women. Notably, the large increase in suicide rates among women in their 20s in 2020 and 2021 may reflect the impact of the COVID-19 pandemic in this context, the lack of support for young women aged 20-30 years. The pandemic appears to have negatively impacted women more than men, with women experiencing mental health issues such as loneliness, depression, anxiety, and posttraumatic stress disorder symptoms, as well as an increased risk of violence against women at home and in the workplace [28]. Loneliness has been reported to be a significant predictor of suicidal thoughts and behavior [29]. A study on deaths before and after the COVID-19 outbreak in Korea [30] reported that the actual number of suicides among women and those under the age of 34 in 2020 significantly exceeded the predictions of suicide rates based on pre-COVID-19 data. This supports the notion that the negative impact of the pandemic on women is associated with increased suicide rates among younger women. Furthermore, the number of Korean women in their 20s and 30s who received medical care for depression in the first half of 2020 increased significantly compared to that in 2019 [31]. Social isolation and loneliness due to social distancing [32] during a pandemic, such as COVID-19, can have a huge impact on mental health, especially for young women, and efforts should be made to ameliorate this.

Drug and carbon monoxide poisoning as a means of suicide among Korean women has steadily increased over the past decade, whereas suicide deaths by pesticide poisoning have decreased significantly. Regulations on the means of suicide, such as the 2012 ban on highly toxic pesticides in Korea [33], appear to have led to a decrease in actual suicide deaths due to pesticide poisoning. Indeed, the restriction of lethal means of suicide is an important component of suicide prevention strategies, as seen in Denmark [34] and Switzerland [35]. The increase in women’s suicide deaths due to drug and carbon monoxide poisoning suggests that women are more likely to choose nondramatic means of suicide as compared to men [36], especially given the ease with which sedatives and sleeping pills can be purchased online as well as charcoal burning that causes carbon monoxide emission. Korea’s Fifth Basic Plan for Suicide Prevention, established in 2023 [33], specifies aims to strengthen the management of risk factors in relation to the means of suicide, including places with a high frequency of suicide, suicide-related media reporting, and restricting dangerous and/or lethal means of suicide. Additionally, gender-specific patterns of suicide means should also be considered.

This study updates Korean women’s suicide death statistics, suicide rate trends, gender- and age-specific suicide characteristics, and suicide means outcomes for the past decade from 2011 to 2021. The results showed that women in their 20s and 30s and women aged 70 years or older were more at risk of suicide deaths in Korea. In addition, there has been a recent increase in drugs and carbon monoxide as the preferred means of suicide for women. Therefore, more sensitive and responsive policies are needed and the following measures should be considered to reduce the suicide rate among women: improve employment; expand mental health promotion programs to mitigate depression; encourage health-promoting behaviors to reduce alcohol consumption and smoking in women; manage women’s preferred means of suicide, and increase sociocultural intolerance of sexual and dating violence.

## Figures and Tables

**Table 1.** The number of suicide death, suicide death rate, and age-standardized suicide death rate (South Korea, 2011 – 2021)

Year	Suicide deaths (person)			Suicide death rate (per 100,000 population)				Age-standardized suicide death rate (per 100,000 standard population)		
	Total	Female	Male	Total	Female	Male	Female:Male	Total	Female	Male
2011	15,906	5,040	10,866	31.7	20.1	43.3	2.2	28.8	17.9	41.1
2012	14,160	4,538	9,622	28.1	18.0	38.2	2.1	25.1	15.6	35.7
2013	14,427	4,367	10,060	28.5	17.3	39.8	2.3	25.1	15.0	36.3
2014	13,836	4,100	9,736	27.3	16.1	38.4	2.4	23.9	14.0	34.6
2015	13,513	3,954	9,559	26.5	15.5	37.5	2.4	22.7	13.3	32.9
2016	13,092	3,849	9,243	25.6	15.0	36.2	2.4	21.9	13.0	31.6
2017	12,463	3,541	8,922	24.3	13.8	34.9	2.5	20.7	12.0	30.0
2018	13,670	3,808	9,862	26.6	14.8	38.5	2.6	22.6	13.0	32.7
2019	13,799	4,069	9,730	26.9	15.8	38.0	2.4	22.6	14.0	31.7
2020	13,195	4,102	9,093	25.7	15.9	35.5	2.2	21.9	14.4	29.9
2021	13,352	4,159	9,193	26.0	16.2	35.9	2.2	22.1	14.7	30.0
2021 compared to 2011 (%)	–16.1	–17.5	–15.4	–18.1	–19.8	–17.1	3.4	–23.0	–17.9	–27.1
2021 compared to 2020 (%)	1.2	1.4	1.1	1.2	1.4	1.2	-0.2	1.1	2.1	0.4

**Table 2.** Women’s suicide rate by age group (South Korea, 2011 – 2021; per 100,000 population)

Year	Age group (year)							
	<20	20–29	30–39	40–49	50–59	60–69	70–79	≥80
2011	2.8	20.0	22.0	19.8	20.7	24.4	43.6	79.6
2012	2.7	15.2	19.8	18.3	17.2	19.9	42.8	73.0
2013	2.3	14.8	20.0	17.8	18.0	18.4	35.4	63.9
2014	1.9	13.4	18.9	17.7	17.4	16.5	32.1	51.2
2015	2.1	12.0	17.9	17.3	14.7	17.4	31.2	50.7
2016	2.3	12.5	17.7	16.5	16.3	14.6	26.5	45.7
2017	1.9	11.4	16.2	16.8	13.7	13.6	23.4	38.7
2018	3.2	13.2	18.3	17.3	15.1	13.6	22.0	37.3
2019	3.5	16.6	20.0	17.1	15.9	14.0	23.5	35.5
2020	3.5	19.3	19.4	17.4	15.1	16.0	17.9	35.2
2021	3.8	19.6	20.7	17.1	16.3	13.1	21.5	31.9
2021 compared to 2011 (%)	37.7	–2.2	–5.7	–13.5	–21.6	–46.1	–50.5	–60.0
2021 compared to 2020 (%)	8.4	1.2	7.0	–1.6	7.3	–18.1	20.3	–9.4

**Table 3.** Women’s suicide rate by means (South Korea, 2011–2021; per 100,000 population)

Year	Suicide means							
	Drugs	Carbon monoxide	Other and unspecified gases	Pesticides	Hanging	Drowning	Fall	Others
2011	0.3	0.6	0.1	3.3	10.0	0.5	4.2	1.2
2012	0.3	0.6	0.2	2.7	8.4	0.6	4.2	1.1
2013	0.3	0.9	0.2	1.8	8.4	0.7	3.8	1.1
2014	0.4	1.3	0.2	1.3	8.0	0.6	3.3	1.0
2015	0.3	1.0	0.4	1.1	7.3	0.6	3.6	1.1
2016	0.5	0.9	0.3	1.2	7.2	0.7	3.2	1.1
2017	0.4	1.1	0.2	1.1	6.6	0.6	3.0	0.9
2018	0.5	1.1	0.2	0.8	7.0	0.6	3.8	0.7
2019	0.6	1.2	0.2	0.9	7.7	0.6	3.7	0.9
2020	0.7	1.1	0.2	0.8	7.7	0.8	3.7	0.8
2021	0.8	1.5	0.2	0.8	7.1	0.8	4.3	0.7
2021 compared to 2011 (%)	220.5	134.3	57.1	–75.0	–28.8	46.9	0.7	–36.1
2021 compared to 2020 (%)	20.6	28.3	–30.0	–1.9	–8.1	–1.9	14.2	–5.9
